# The 50 most influential papers pertaining to the Ilizarov method: A bibliometric analysis

**DOI:** 10.1016/j.jor.2022.02.010

**Published:** 2022-02-11

**Authors:** Ben Murphy, Shane Irwin, Finbarr Condon

**Affiliations:** a University Hospital Limerick, Dooradoyle, Limerick, Ireland; b Croom Orthopaedic Hospital, Croom, Limerick, Ireland

**Keywords:** Ilizarov method, Distraction osteogenesis, Citation, Bibliometric analysis, Orthopedic surgery

## Abstract

The Ilizarov method has become a widely recognised surgical technique. A bibliometric analysis of the 50 most-cited publications relating to the Ilizarov method was carried out. Cumulative number of citations was 4,918. Mean number of citations was 98. h-index was 50. Impact factor of these journals ranged from 0.5-5.082. Our study suggests that a highly cited and influential paper likely originated from an American journal with a high impact factor and was published in the 1990s/2000s. Our compilation of the 50 most influential papers on the Ilizarov method will prove invaluable to those in training and those involved in further advancing the technique.

## 1 Introduction

Prof Gavriil Ilizarov's career began treating returning World War II soldiers. Famously, his method of distraction osteogenesis for bone lengthening was discovered by accident when callus was seen in a subject who distracted his frame instead of the usual compression.^1^ Ilizarov's method made its first appearance in peer-reviewed journals almost 40 years ago.^2^ The peak of its application, outside of its well-known usage in Russia, came during the 1990s.^3^ As we will see in this study, the 1990s also coincided with the peak of published papers on its use.^3^ As a testament to its success in bone repair and reconstruction, we have seen in the range of 50–100 articles on its various uses published each year.^4^ It offers an alternative solution to bone conditions that may otherwise have been solved with more drastic measures such as amputation.^5^, ^6^, ^7^ Despite the degree of expertise needed to perform correctly and its steep learning curve, it has gained popularity in many countries.

Our understanding of this technique has grown with its continued use and multiple publications. We stand to learn much from those that have gone before us with this technique and their experiences in the published literature are a crucial part of advancements in the field. An in-depth look and subsequent ranking of these publications allows us to ascertain which papers have had the most influence in terms of our clinical decision-making.

Citations are a form of recognition between authors of the impact that a colleague's work has had on their own research. Looking at how frequently an article is cited, we can evaluate the impact that article has had on the progression of the field in question. Citations are used in the metric for determining an article's impact factor (IF). We are drawn to journals with a higher impact factor and so citations can be said to indirectly influence the journals we read. IF is equal to the total number of citations for all publications in a journal over the past year divided by the total number of publications published in that journal in the previous two years.^8^

Other authors have sought to find the most influential papers across a broad range of surgical specialties including orthopaedics,^9^ plastics,^10^ breast surgery,^11^ general surgery^12^ and urology.^13^ Within the discipline of orthopaedics, there have been citation analyses published in the areas of hip and knee arthroplasty,^14^ shoulder surgery,^15^ spinal deformity surgery^16^ and foot and ankle.^17^ A bibliometric analysis of all articles containing the word “bone lengthening” was published in 2019. However, this paper dealt with other methods of bone lengthening, not solely the Ilizarov method. It also only included papers published from 2001 to 2017.^18^ To our knowledge, this is the first study examining the most influential papers relating to the Ilizarov method.

**Fig. 1:**
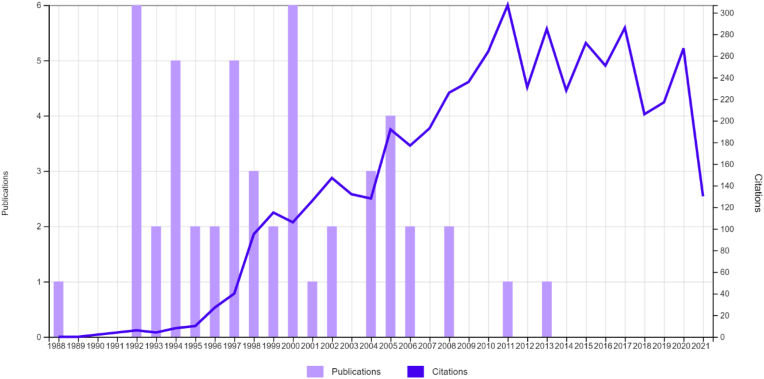
Graph showing trend of publications and citations.

Our aim with this study was to discover the 50 most-cited papers pertaining to the Ilizarov method by carrying out a bibliometric analysis of previous publications using the Web of Science database (see Fig. 1).

## 2 Methods

**Fig. 2:**
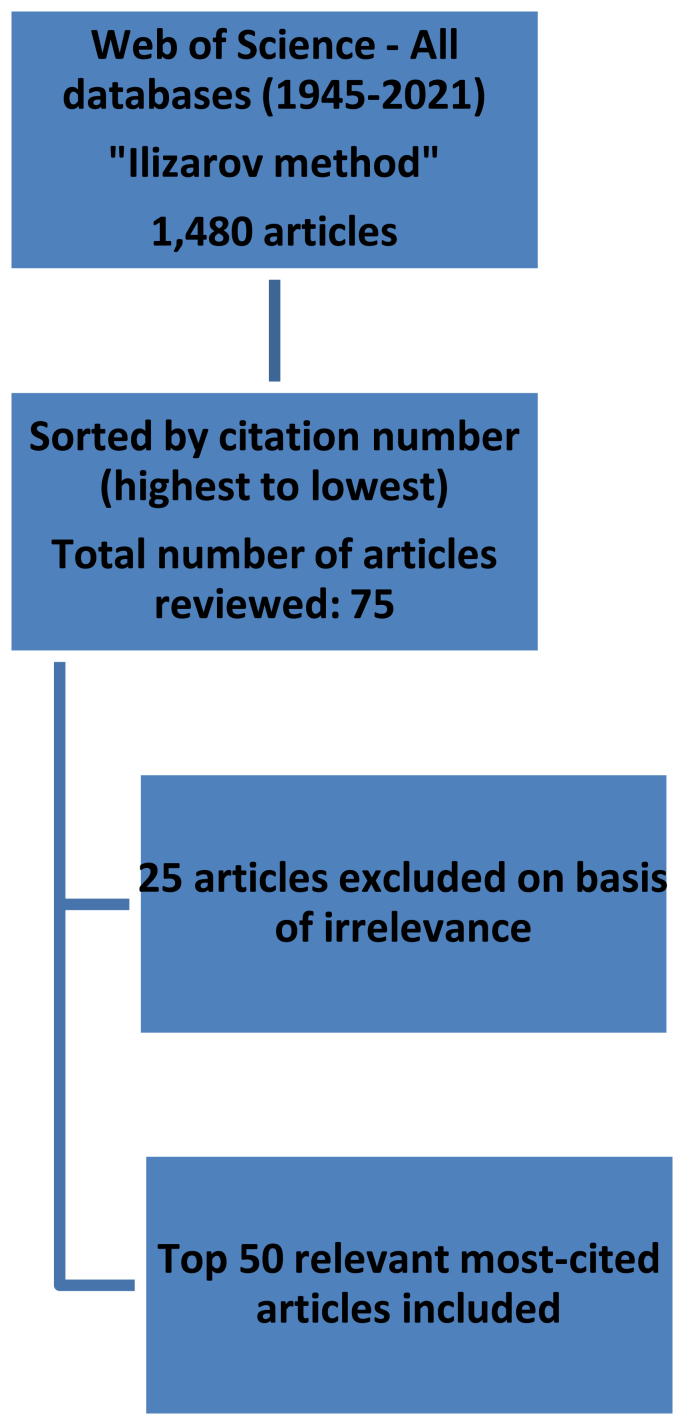
Search strategy for bibliometric analysis.

To examine the top 50 articles regarding the Ilizarov method, the Web of Science Collection search engine was employed in August 2021. A single search term, “Ilizarov method” was used and articles from 1945 to 2021 were examined. There were no limitations applied to our search filter. 1,480 papers were recovered on the initial search. These papers were then ordered from most citations to least number of citations. Two independent authors reviewed the results and complete agreement between authors was required for an article to be included. If an abstract did not yield sufficient information to decide on article inclusion, a full-text review was carried out. Papers deemed not relevant to the Ilizarov method were excluded. The first 75 papers were examined thoroughly. This led to the exclusion of 25 papers based on irrelevance to the topic in question (Fig. 2). A decision was made to conclude our search at 75 papers as after that, the number of citations per paper did not fall dramatically. To illustrate this point, the difference in citations between the 75th and the 100th paper was 7. The remaining 50 most-cited papers were put into tables. Further analysis of these papers was carried out based on various factors: mean citation number, the year of publication, publishing institution, country of origin and what journal it was published in. Publication year was divided into decades. The calculation for mean citation number involved dividing the number of citations an article has by the number of years that had passed since it was published.

## 3 Results

The cumulative total of citations for the most-cited 50 publications was 4,918. Each paper was cited 98 times on average. The h-index of this list of articles was 50. The top 50 articles can be found in Table 1. The most-cited paper was by Gavriil Ilizarov himself with 428 citations, published in 1988.^19^ The 50th paper was by Birch^20^ and was cited 52 times. The oldest paper was published in 1988, again this was Ilizarov's above-mentioned paper “The principles of the Ilizarov method”.^19^ The most recent publication came from Papakostidis et al.^21^ in the Bone and Joint Journal in 2013, who performed a systematic review of the Ilizarov method in patients with long bone defects.

**Table 1:** Top 50 cited articles relevant to Ilizarov method.

Title	Authors	Total citations	Mean citation number
The principles of the Ilizarov method	Ilizarov, Ga	428	12.59
Ilizarov bone transport treatment for tibial defects	Paley, D; Maar, DC	224	10.18
Limb-lengthening, skeletal reconstruction, and bone transport with the Ilizarov method	Aronson, J; Rock, L	223	8.92
Le Fort III advancement with gradual distraction using internal devices	Chin, M; Toth, BA	182	7.28
Mechanical axis deviation of the lower-limbs - preoperative planning of uniapical angular deformities of the tibia or femur	Paley, D; Tetsworth, K	180	6
The treatment of infected nonunions and segmental defects of the tibia by the methods of Ilizarov	Cattaneo, R; Catagni, M; Johnson, Ee	156	5.2
Complications of limb lengthening - a learning-curve	Dahl, Mt; Gulli, B; Berg, T	152	5.43
Experimental and clinical experience with distraction osteogenesis	Aronson, J	135	4.82
Treatment of high-energy tibial plateau fractures by the Ilizarov circular fixator	Dendrinos, GK; Kontos, S; Katsenis, D; Dalas, A	126	4.85
Segmental tibial defects - comparing conventional and Ilizarov methodologies	Cierny, G; Zorn, Ke	117	4.18
Treatment of congenital pseudarthrosis of the tibia using the Ilizarov technique	Paley, D; Catagni, M; Argnani, F; Prevot, J; Bell, D; Armstrong, P	112	3.73
Skeletal defects - a comparison of bone-grafting and bone transport for segmental skeletal defects	Green, Sa	109	3.89
The Ilizarov method in nonunion, malunion and infection of fractures	Marsh, DR; Shah, S; Elliott, J; Kurdy, N	108	4.32
Management of segmental defects by the Ilizarov intercalary bone transport method	Green, Sa; Jackson, Jm; Wall, Dm; Marinow, H; Ishkanian, J	106	3.53
Simultaneous treatment of tibial bone and soft-tissue defects with the Ilizarov method	Rozbruch, SR; Weitzman, AM; Watson, JT; Freudigman, P; Katz, HV; Ilizarov, S	102	6.38
The correction of complex foot deformities using Ilizarov distraction osteotomies	Paley, D	100	3.45
Mechanical axis deviation of the lower-limbs - preoperative planning of multiapical frontal plane angular and bowing deformities of the femur and tibia	Paley, D; Tetsworth, K	100	3.33
Lengthening of the hypoplastic mandible by gradual distraction in childhood - a preliminary-report	Klein, C; Howaldt, Hp	99	3.67
Complications of use of the Ilizarov technique in the correction of limb deformities in children	Velazquez, Rj; Bell, Df; Armstrong, Pf; Babyn, P; Tibshirani, R	97	3.34
Bifocal compression-distraction in the acute treatment of grade III open tibia fractures with bone and soft-tissue loss - a report of 24 cases	Sen, C; Kocaoglu, M; Eralp, L; Gulsen, M; Cinar, M	94	5.22
Hinged Ilizarov external fixation for correction of antebrachial deformities	Marcellin-Little, DJ; Ferretti, A; Roe, SC; DeYoung, DJ	88	3.67
Repair of tibial nonunions and bone defects with the Taylor Spatial Frame	Rozbruch, S. Robert; Pugsley, Jacob S.; Fragomen, Austin T.; Ilizarov, Svetlana	82	5.86
Treatment approaches for congenital pseudarthrosis of tibia: results of the EPOS Multicenter Study	Grill, F; Bollini, G; Dungl, P; Fixsen, J; Hefti, F; Ippolito, E; Romanus, B; Tudisco, C; Wientroub, S	82	3.73
Tibial bone defects treated by internal bone transport using the Ilizarov method	Song, HR; Cho, SH; Koo, KH; Jeong, ST; Park, YJ; Ko, JH	80	3.33
The replacement of long tubular bone defects by lengthening distraction osteotomy of one of the fragments	Ilizarov, Ga; Ledyaev, Vi	80	2.67
Distraction osteogenesis in the treatment of long bone defects of the lower limbs effectiveness, complications and clinical results; a systematic review and meta-analysis	Papakostidis, C.; Bhandari, M.; Giannoudis, P. V.	77	8.56
The Ilizarov method in infected nonunion of fractures	Maini, L; Chadha, M; Vishwanath, J; Kapoor, S; Mehtani, A; Dhaon, BK	76	3.45
Nonunion of the humerus after failure of surgical treatment - management using the Ilizarov circular fixator	Patel, VR; Menon, DK; Pool, RD; Simonis, RB	76	3.45
Joint distraction in treatment of osteoarthritis - (II): effects on cartilage in a canine model	van Valburg, AA; van Roermund, PM; Marijnissen, ACA; Wenting, MJG; Verbout, AJ; Lafeber, FPJG; Bijlsma, JWJ	68	3.09
Joint distraction in treatment of osteoarthritis: a two-year follow-up of the ankle	van Valburg, AA; van Roermund, PM; Marijnissen, ACA; van Melkebeek, J; Lammens, J; Verbout, AJ; Lafeber, FPJG; Bijlsma, JWJ	67	2.91
Mandibular lengthening by external distraction - an experimental-study in the rabbit	Califano, L; Cortese, A; Zupi, A; Tajana, G	67	2.39
Management of complex tibial and femoral nonunion using the Ilizarov technique, and its cost implications	Patil, S.; Montgomery, R.	66	4.13
Treatment of malunion and nonunion at the site of an ankle fusion with the Ilizarov apparatus	Katsenis, D; Bhave, A; Paley, D; Herzenberg, JE	66	3.88
Interfragmentary motion in tibial osteotomies stabilized with ring fixators	Duda, GN; Sollmann, M; Sporrer, S; Hoffmann, JE; Kassi, JP; Khodadadyan, C; Raschke, M	66	3.3
Subchondral bone remodeling is related to clinical improvement after joint distraction in the treatment of ankle osteoarthritis	Intema, F.; Thomas, T. P.; Anderson, D. D.; Elkins, J. M.; Browns, T. D.; Amendola, A.; Lafeber, F. P. J. G.; Saltzman, C. L.	64	5.82
Treatment of congenital pseudarthrosis of the tibia - a multicenter study in Japan	Ohnishi, I; Sato, W; Matsuyama, J; Yajima, H; Haga, N; Kamegaya, M; Minami, A; Sato, M; Yoshino, S; Oki, T; Nakamura, K	62	3.65
The effect of low-intensity pulsed ultrasound on callus maturation in tibial distraction osteogenesis	El-Mowafi, H; Mohsen, M	61	3.59
Arthrodesis of the knee after an infected arthroplasty using the Ilizarov method	Oostenbroek, HJ; van Roermund, PM	61	2.9
Prolonged clinical benefit from joint distraction in the treatment of ankle osteoarthritis	Ploegmakers, JJW; van Roermund, PM; van Melkebeek, J; Lammens, J; Bijlsma, JWJ; Lafeber, FPJG; Marijnissen, ACA	60	3.53
Fractures after femoral lengthening using the Ilizarov method	Danziger, Mb; Kumar, A; Deweese, J	60	2.22
The Ilizarov method in the management of relapsed club feet	Bradish, CF; Noor, S	59	2.68
Lengthening of the tibia over an intramedullary nail, using the Ilizarov external fixator - major complications and slow consolidation in 9 lengthenings	Kristiansen, LP; Steen, H	59	2.57
The use of frozen-allograft radial head replacement for treatment of established symptomatic proximal translation of the radius: preliminary experience in five cases	Szabo, RM; Hotchkiss, RN; Slater, RR	59	2.36
Results of the Wagner and Ilizarov methods of limb-lengthening	Aaron, AD; Eilert, RE	58	2.23
Bone transport in the management of post-traumatic bone defects in the lower extremity	Mekhail, AO; Abraham, E; Gruber, B; Gonzalez, M	56	3.11
Treatment of nonunion of the humerus using the Ilizarov external fixator	Lammens, J; Bauduin, G; Driesen, R; Moens, P; Stuyck, J; De Smet, L; Fabry, G	56	2.33
Congenital pseudarthrosis of the tibia associated with neurofibromatosis-1: treatment with Ilizarov's device	Boero, S; Catagni, M; Donzelli, O; Facchini, R; Frediani, PV	54	2.16
A retrospective analysis of comminuted intra-articular fractures of the tibial plafond: open reduction and internal fixation versus external Ilizarov fixation	Bacon, Stacy; Smith, Wade R.; Morgan, Steven J.; Hasenboehler, Erik; Philips, Giby; Williams, Allison; Ziran, Bruce H.; Stahel, Philip F.	53	3.79
Salvage of infected total knee fusion: the last option	Wiedel, JA	53	2.65
Use of the Ilizarov method to correct lower limb deformities in children and adolescents	Birch, JG; Samchukov, ML	52	2.89

The 1990s saw the greatest number of papers (n = 27). The 20 year period between 1990 and 2010 made up 94% of the top 50 papers (Table 2). These 50 papers were published in 20 unique journals. The impact factors of these journals ranged from 0.5 to 5.082. The language of publication in all instances was English. These publications represented several research areas including orthopaedics, general surgery, paediatrics, rheumatology, sport sciences and maxillofacial surgery. *Clinical Orthopaedics & Related Research* (n = 13) produced the highest number of papers on the list – followed by *Journal of Bone & Joint Surgery – British Volume* (n = 6), *Journal of Bone & Joint Surgery – American Volume* (n = 4), *Journal of Orthopaedic Trauma* (n = 4) and *Osteoarthritis & Cartilage* (n = 4). The definitive list of journals can be found in Table 3.

**Table 2:** Top 50 articles published by decade.

Decade	Number of articles published
2010's	2
2000's	20
1990's	27
1980's	1

**Table 3:** Top 50 cited articles publication location & impact factor.

Journal	Number	Impact Factor (2021)
Clinical Orthopaedics & Related Research	13	4.329
Journal of Bone & Joint Surgery – British Volume	6	4.306
Journal of Bone & Joint Surgery – American Volume	4	4.578
Journal of Orthopaedic Trauma	4	2.512
Osteoarthritis & Cartilage	4	4.793
Journal of Paediatric Orthopaedics	3	1.909
Injury	2	2.106
International Orthopaedics	2	2.854
Acta Orthopaedica Scandinavica	1	2.965
Bone and Joint Journal	1	5.082
Bulletin of the Hospital for Joint Diseases Institute	1	0.5
Cleft Palate Craniofacial Journal	1	1.347
Journal of Cranio Maxillo Facial Surgery	1	1.766
Journal of Hand Surgery – American volume	1	2.124
Journal of Oral & Maxillofacial surgery	1	1.781
Journal of Paediatric Orthopaedics, Part B	1	0.832
Journal of the American Academy of Orthopaedic Surgeons	1	2.286
Journal of Trauma, Injury, Infection & Critical Care	1	2.961
Plastic & Reconstructive Surgery	1	4.73
Veterinary Surgery	1	1.255

10 unique countries contributed to the top 50. USA provided the most articles with 24 (48%), followed by Italy with 5 (10%) and Netherlands with 5 (10%). A full list of countries of origin can be found in Table 4. 10 different authors provided the top 50 articles. All authors publishing 2 or more papers can be found in Table 5. Paley appeared most frequently as first author with 6 (12%). This was closely followed by Lafeber and Von Roermund with 4 papers each (8%). Gavriil Ilizarov himself authored 2 papers (4%).

**Table 4:** Countries of origin of top 50 cited articles.

Country	Number of articles
USA	26 (48%)
Italy	5 (10%)
Netherlands	5 (10%)
England	4 (8%)
Belgium	3 (6%)
Germany	2 (4%)
Greece	2 (4%)
Austria	1 (2%)
Canada	1 (2%)
Egypt	1 (2%)

**Table 5:** First authors who published >1 article and number of articles published.

Author	Number of articles
Paley	6
Lafeber	4
Van Roermund	4
Bijlsma	3
Catagni	3
Lammens	3
Marijnissen	3
Aronson	2
Green	2
Ilizarov	2

## 4 Discussion

In our study,we searched the Web of Science database to find the 50 most-cited papers on the Ilizarov method. In this way, we hoped to establish the papers which had influenced the field. A citation analysis such as this one, is important to the wider orthopaedic community as it chronicles the papers, authors, journals and eras that have shaped the discipline as we know it. Our study provides insight into the practice-changing developments that have taken place over the past 76 years.

In our study, citation number was used to estimate the influence of a certain paper and warrant its inclusion in the top 75. This method of citation analysis has been debated by some,^22^ and does carry limitations which have been discussed by previous authors.^10^ What it does allow for however, is a gauge of peer-recognition and allows us to examine the readership of an article. One must remember that number of citations does not directly correspond with study quality. For example, poorly-designed studies (by present day standards), may have a higher number of citations than a more recent study purely because it has had more time to accrue citations. If an article does have a high number of citations, it indicates that other researchers found its content useful. IF, a measure of citations, plays a vital role in how we interpret research and so these indicators are at the forefront of everyone's mind when critically analysing any piece of research.

The most-cited paper was “The principles of the Ilizarov method”^19^ (1988) published by Ilizarov himself. This paper has been cited on 428 occasions. Its average citation number is 12.59. This article is based on a speech he delivered at the Annual Scientific Program of the Alumni association, supplemented by material he previously presented at a three-day international conference on the Ilizarov techniques for managing problematic skeletal issues.

The second most-cited paper was Paley's “Ilizarov bone transport treatment for tibial defects”^23^ which was published in the Journal of Orthopaedic Trauma in 2000. His article was cited a total of 224 times with a mean citation number of 10.18. He conducted a retrospective case series of 19 patients with tibial defects treated with the Ilizarov method. All patients went on to achieve union. He found that his results compared favourably to other studies looking at other methods of bone grafting and even other studies concerning the Ilizarov method itself, particularly taking into account the size of the defect in his patients. While this paper has fewer citations in terms of absolute number than number one on our list, it is important to acknowledge that it was published 12 years later and its mean citation number (10.18) is quite respectable compared to 12.59 for the top-cited paper. Ilizarov's paper also dealt with the underlying principles of the technique and so would be relevant to a wider audience whereas Paley's only looked at the technique in the context of tibial defects. However, his contribution to the field of limb lengthening and reconstruction is evident as he was first author on the highest number (n = 6) of papers in this study (Table 5).

10 unique countries contributed to the top 50, with the USA contributing the majority (Table 4). That the USA contributed the highest number of papers should come as no surprise, as this has been reflected in other studies of a similar nature.^24^^,^^25^ This finding also is in keeping with the high volume of orthopaedic clinical and academic activity exhibited by the USA. The 1990s and 2000s were the most prolific time periods with 94% (n = 47) of papers published in this era (Table 2). 20 unique journals contributed to the top 50 (Table 3). An American journal, Clinical Orthopaedics & Related Research, had the highest number of publications with 13 (Table 3), again illustrating the influence that American studies have had on the field. Indeed, 4 out of the top 5 journals in terms of absolute publication number are American. Barring Ilizarov's paper at number 1, papers 1–5 on the list came from prestigious journals with IF in the range of 2.512–4.73. Our research would suggest that for a paper on the Ilizarov method to be highly-cited and influential, it likely originated from an American journal, with a high impact factor and was published in the 1990s/2000s.

There are several inherent limitations to a bibliometric analysis such as ours. This has been detailed and acknowledged by previous authors carrying out a similar study type.^16^^,^^26^ The Web of Science Collection search engine extends from the present day as far back as 1945. For this reason, any articles published before then are automatically excluded and there is a real possibility of excluding some of the “classic” papers on the Ilizarov method. Previous citation analyses in other areas placed limits on their search. There were no restrictions on our search and the authors felt this reduced the possibility of excluding potentially relevant articles. It has been alluded to in previous studies that more recent papers (that may be of a potentially higher quality) are disadvantaged as the “classic” papers have been around for longer and thus have had greater opportunity for citation. Our use of the mean citation number is our attempt to control for this and to allow us to make a meaningful comparison between contemporary and historic papers.

Aspects of a bibliometric analysis make it difficult to control for bias, as was the case in our study. Self-citation, language bias, journal bias and in-house bias (among others) were not controlled for in this study.^27^ There are several phenomena that can influence the results of a bibliometric analysis. *Obliteration by incorporation* is one of these. This refers to the highly-influential or “classic” articles becoming incorporated into clinical practice and widely-acknowledged and so, the need to cite them no longer remains.^28^ *Incomplete citing* may also occur within papers if an author is attempting to persuade their audience instead of acknowledging a previous piece of work.^29^

## 5 Conclusion

Our study highlights the 50 most-cited papers pertaining to the Ilizarov method. In this way, we have established those papers which have influenced the development and implementation of the technique. We have seen the journals, countries and authors that have made the greatest contribution. We are aware that a citation analysis does not directly measure scientific quality but it does indicate those papers which have significantly contributed to the area of interest. This compilation of influential papers will prove invaluable to orthopaedic trainees along with those involved in advancing the Ilizarov method as a surgical technique.

## Funding

“This research did not receive any specific grant from funding agencies in the public, commercial, or not-for-profit sectors.”

## Declaration of competing interest

“The authors have no conflicts of interest to disclose.”
